# The International Medical Congress

**Published:** 1887-06

**Authors:** 


### THE INTERNATIONAL MEDICAL CONGRESS.

This body is appointed to meet in Washington City on September —, 1887. It is earnestly hoped that the Congress will be all that in its incipiency was hoped for it. No consideration should prevent the great and true representatives of the profession in the United States from a full and cordial co operation in the Congress and from a zealous support to the great objects in view. There is one point which overrides every other, and which should exert a paramount influence upon the minds and hearts of the profession, and that is the National pride which every medical man should feel, and it seems to us must feel, in the success of this great Medical Congress of Nations, for the first time appointed to meet on American soil—at the centre and Capital of the Nation where the tree of Liberty was planted, and in which the American Eagle, the emblem of strength and power, with hovering and protecting wings, perches perpetually. The Spirit of Patriotism and true Hospitality is surely not wanting in the hearts of American physicians who, in times past, have ever maintained a high position before the world for the love of Country not less than for the principles of their noble profession. It would seem that it should inspire the highest National spirit and the warmest professional love and hospitality to think of the coming of the great lights of the profession from other countries to shake hands with us upon our own soil, at our own National Capital, that we may extend to them that warm social greeting and true professional brotherhood for which medical men are noted the world over.

We are glad to say that we have received many assurances from abroad, and from representative men in the profession at home, that the Congress will be a great success, and that the National reputation and the patriotic pride of the American profession will be well maintained.

